# A systematic approach for evaluating the role of surface-exposed loops in trypsin-like serine proteases applied to the 170 loop in coagulation factor VIIa

**DOI:** 10.1038/s41598-022-07620-7

**Published:** 2022-03-08

**Authors:** Anders B. Sorensen, Per Jr. Greisen, Jesper J. Madsen, Jacob Lund, Gorm Andersen, Pernille G. Wulff-Larsen, Anette A. Pedersen, Prafull S. Gandhi, Michael T. Overgaard, Henrik Østergaard, Ole H. Olsen

**Affiliations:** 1 Global Research, Novo Nordisk A/S, 2760 Måløv, Denmark; 2 Department of Chemistry and Bioscience, Aalborg University, 9220 Ålborg, Denmark; 3 Global and Planetary Health, College of Public Health, University of South Florida, Tampa, FL 33612 USA; 4 Department of Molecular Medicine, Morsani College of Medicine, University of South Florida, Tampa, FL 33612 USA; 5 Present Address: Novo Nordisk Foundation Center for Basic Metabolic Research, Section for Metabolic Receptology, University of Copenhagen, Blegdamsvej 3b, 2200 Copenhagen, Denmark

**Keywords:** Proteases, Proteolysis

## Abstract

Proteases play a major role in many vital physiological processes. Trypsin-like serine proteases (TLPs), in particular, are paramount in proteolytic cascade systems such as blood coagulation and complement activation. The structural topology of TLPs is highly conserved, with the trypsin fold comprising two β-barrels connected by a number of variable surface-exposed loops that provide a surprising capacity for functional diversity and substrate specificity. To expand our understanding of the roles these loops play in substrate and co-factor interactions, we employ a systematic methodology akin to the natural truncations and insertions observed through evolution of TLPs. The approach explores a larger deletion space than classical random or directed mutagenesis. Using FVIIa as a model system, deletions of 1–7 amino acids through the surface exposed 170 loop, a vital allosteric regulator, was introduced. All variants were extensively evaluated by established functional assays and computational loop modelling with Rosetta. The approach revealed detailed structural and functional insights recapitulation and expanding on the main findings in relation to 170 loop functions elucidated over several decades using more cumbersome crystallization and single deletion/mutation methodologies. The larger deletion space was key in capturing the most active variant, which unexpectedly had a six-amino acid truncation. This variant would have remained undiscovered if only 2–3 deletions were considered, supporting the usefulness of the methodology in general protease engineering approaches. Our findings shed further light on the complex role that surface-exposed loops play in TLP function and supports the important role of loop length in the regulation and fine-tunning of enzymatic function throughout evolution.

## Introduction

**Figure 1:**
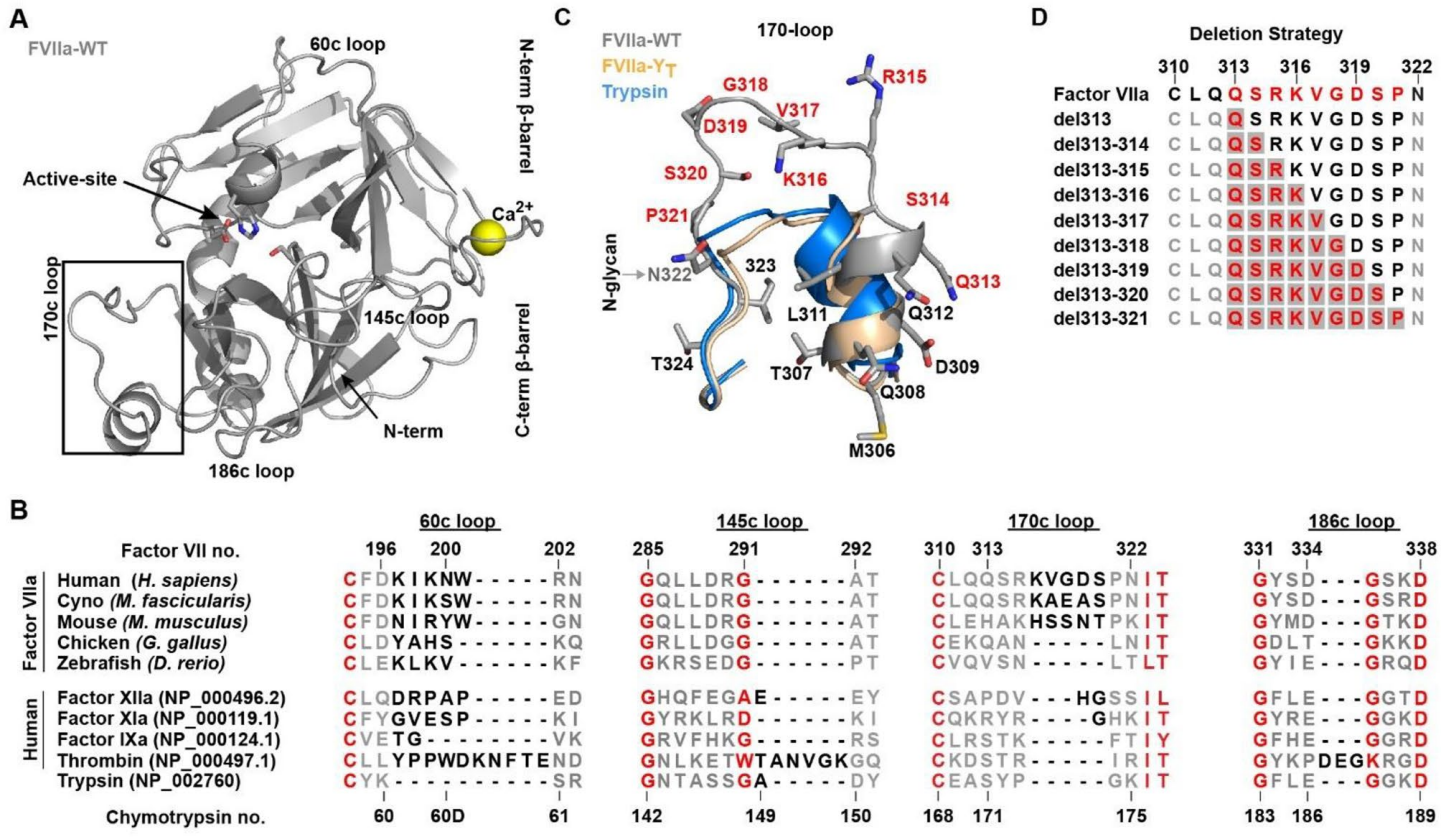
FVII Structural Features and the Evolution of Surface-Exposed Loops. (**A**) Crystal structure of FVIIa:sTF (PDB ID:1dan, grey ^21^), shown in standard representation. (**B**) Alignment of serine proteases from various stages of blood coagulation compared with the ancestral protease trypsin, together with the surface-exposed loops of FVIIa from various species (see supplemental Fig. S1). Conserved residues are shown in red, non-conserved in grey and the loop extension region in black. Both numbering based on the human Factor VII sequence and on the consensus Chymotrypsin numbering is shown. (**C**) The different conformations of the 170 loop in FVIIa, FVIIa-Y_T_ (4z6a, wheat ^25^) and trypsin (3ptn, blue ^74^) with deletion scanned residues in red and an N-glycan attachment site. (**D**) Deletion strategy in FVIIa with scanned residues in red and start positions for each deletion window marked with grey. A total of 39 variants were generated by moving the deletion windows through the loop.

Trypsin-like serine protases (TPLs) play a pivotal role in a wide range of biological functions by mediating cellular signalling through highly regulated and limited proteolysis^1–3^. Proteases with the trypsin fold are defined by their hallmark double β-barrel topology connected by several surface-exposed loops of distinct functional relevance (Fig. 1A)^4,5^. While the N- and C-terminal β-barrels are highly conserved and serve as the scaffold for active-site triad placement and substrate cleft formation, functional diversity and specificity of substrate interactions are mainly mediated by the surface-exposed loops^2,6^. These loops have been adapted extensively in length and amino acid composition (Fig. 1B and Sup Fig. 1) through the TLP linage, allowing for a high level of evolutionary divergence within the family^3,7,8^. This has facilitated diversified functions for TLPs ranging from immunity and coagulation to metabolism, with an impressive diversity in regulatory mechanisms^2,9^ governed by a number of co-factors and exo-sites interactions^9–11^. A prime example is blood coagulation, where a number of TLPs act in a co-factor-dependent enzymatic cascade on the membrane surface to halt bleeding in an organized fashion^12–15^.

Remarkable insights into the detailed structure–function relationship of TLPs has been gathered over the last decades increasing our appreciation of how surface-exposed loops on the protease domain can lead to specific functional characteristics. For coagulation factor VIIa (FVIIa), the 170 loop has proven itself to be of predominant importance in the co-factor-mediated initiating of coagulation and as such has received considerable attention^16–20^. Several crystal structures are available which^21–26^, in combination with extensive molecular dynamics simulations, has been used to explore the TLP domain ensemble and different loop conformations^27–35^, shedding light on allostery, substrate recognition and co-factor interaction. Past work has substantiated that the conformation of the 170 loop 1) plays a central role in co-factor mediated allosteric regulation of FVIIa activity by Tissue Factor (TF) 2) is involved in maintaining free FVIIa in its low activity state and 3) influences the initiation of coagulation upon TF binding^16^. From an evolutionary perspective, the 170 loop of human FVIIa has been extended by 3–5 residues and a N-glycan has been added when compared to ancestral trypsin and paralogous TLPs in the coagulation cascade (Fig. 1B and C)^36^. This loop extension is likely to have occurred after the divergence of the mammalian line as it is not present in lower vertebrates such as fish or bird (Fig. 1B)^37,38^, and may have developed due to selection pressure for a more tightly controlled coagulation system^39^.

Elucidation of such detailed understanding of surface loop functions in TLPs from structural evidence requires cumbersome crystallization trials and data collection. Even if crystallization trials are successful, the loops may not be resolved in the density due to their flexible nature or they may be distorted by crystal contacts, reducing the insights from such studies^23,40–43^. The insights derived from extensive studies using grafting methods and various point mutation are unquestionably valuable^44–48^; however, such results can be confounded and their interpretation unclear since both the position of a residue in relation to the remainder of the loop and relative to the protease body may affect activity and specificity. More extensive or high-throughput approaches to investigate the function of TLPs^49^, or re-engineer new functions, often rely on random or direct mutagenesis, but due to the likely deleterious effects of introducing truncations above 2–3 amino acids at many locations, such methods only sample a limited space when it comes to the effects of loop length on protease function. Furthermore, local deletion mutations may be considered more destructive than a corresponding insertion at the same position as the protein backbone is more constrained; insertions can sometimes be accommodated by “looping out” segments^50^. Other approaches that work on global protein truncations, such as incremental truncation and combinatorial recombination^51^, may be less useful for engineering TLP function since the palette of characteristics are associated with the behaviour of distinct surface-exposed loops^44,52–54^.

Here, we investigate the applicability of a systematic approach inspired by the evolutionary “staircase” (whereby the length of a given loop can vary greatly between species in distinct “steps”) as observed for TPLs and even between paralogs within the same signalling cascade (Fig. 1 B). The 170 loop from FVIIa was chosen as a model system due to its crucial involvement in activity, specificity and allosteric control of this protease. Using a deletion window size of 1 to 7 residues through positions 313{c170A} to 321{c170I} (FVIIa full-length numbering is used in the remaining part of the article; chymotrypsin numbers are indicated in curly brackets) incrementally proceeding through the entire loop from the N to C-terminus direction, generated a total of 39 variants that were fully reconstructed in silico, recombinantly produced and experimentally characterized in the present study (Fig. 1D). We found that the combined methodology of experimental characterization and *in-silico* loop reconstruction with the Next-generation Kinematic Closure (NGK) protocol^55^ allowed for detailed structure–function insights. Importantly, our investigation recapitulates the main features described previously for 170 loop function in FVIIa. In addition, the approach revealed that the variant with highest amidolytic activity was achieved by a six-amino acid truncation – i.e. shorter than the shortest naturally appearing 170 loop variant – and as such would not have been detected with the sampling space of directed/random mutagenesis.

## Experimental procedures

### Materials

S-2288 (D-Ile-Pro-Arg-pNA) and S-2765 (Z-D-Arg-Gly-Arg-pNA) were purchased from Chromogenix (Mölndal, SWE), pABA (4-amino-benzamidine) was from Sigma-Aldrich (GER). All other chemicals were from Sigma Aldrich (GER) or Merck AG (GER) and of analytical grade or highest quality commercially available. Recombinant human FVIIa was prepared as described previously^56^. Recombinant human soluble tissue factor 1–219 (sTF) was prepared as described previously^57^, except that a reductase-deficient *E. coli* strain BL21 Origami (Novagen, GER) was used. FXa for activation purposes was purchased from Molecular Innovations (US) and coupled to CNBr-Sepharose 4B FF using the manufacturer’s instructions (GE Healthcare, US).

### Protein mutagenesis and expression

Human wild-type FVII cDNA was cloned into a QMCF vector (Icosagen AS, ES) and all variants were generated using a PCR-based site-directed mutagenesis method with KOD Xtreme Hot Start DNA Polymerase (Novagen, US) according to manufacturer’s instructions. Introduction of the desired mutations were verified by DNA sequencing of the entire FVII cDNA region (MWG Biotech, GER). The QMCF Technology, a semi-stable episomal mammalian expression system obtained from Icosagen AS (ES), was used for expression of the FVII variants in a QMCF CHO cell line (CHO-EBNALT85) and cells were cultivated according to manufacturer’s instructions. During a period of 2–3 weeks, the transfected cell cultures were expanded to 0.3–1 L and the media harvested by centrifugation and 0.22 μm filtration. The 39 variants were successfully expressed using a CHO cell line with an observable decrease in yield for variants with significantly shortened loops.

### Protein purification and verification

For all FVII variants, the expression medium was adjusted to pH 7.0 and CaCl_2_ was added to 5 mM. Purification was performed using an Äkta Xpress system (GE Healthcare, US) with a Gla-domain specific immunoaffinity purification step^36^ performed essentially as described previously^58^ with a 10 mM histidine buffer at pH 6.0. Protein was eluted directly onto a custom-packed SourceQ15 (GE Healthcare, US) column with a 10 mM histidine pH 6.0, 15 mM EDTA, 0.005% (v/v) Tween-80 buffer. A wash step with 10 mM histidine pH 6.0, 150 mM NaCl, and 0.005% (v/v) Tween-80 removed excess EDTA, and elution was performed using 10 mM histidine pH 6.0, 200 mM NaCl, 10 mM CaCl_2_ and 0.005% (v/v) Tween-80. Activation was performed by passing the protein solution through a custom-packed Tricon column (GE Healthcare, US) with FXa coupled to Sepharose 4B FF at 18 °C; the mean residence time was 1–2 h. Alternatively, a batch activation approach was used with the same resin for 5–24 h at room temperature. In general the variants were activated to > 90%, with certain variants only activating to ~ 50% (variants 1, 2, 3, 4, 18, 26, 27, 29, 34 and 36) as shown on a Novex 4–12% SDS-PAGE (Life Technologies, US), likely caused by the activation event being partially driven by an auto-catalytic mechanism^59^. The amount of active protein was determined under reductive conditions with a X-Bridge C4 reverse phase (RP) column (Waters, US) on an Alliance HPLC with a 474 fluorescence detector (Waters, US) and separating the protein into heavy and light chains was followed by integration of the fluorescence signal contained in the light-chain peak. A recombinant FVIIa (Novo Nordisk A/S, DK) standard curve was used to calculate the amount of protein from the integrated peak.

### Tissue factor binding, amidolytic activity and small molecule inhibition assays

All assays were carried out in 50 mM Hepes pH 7.4, 100 mM NaCl, 10 mM CaCl_2_, 0.01% Tween-20 (assay buffer) with sample preparation and dilution performed using a STARlet liquid handling robot (Hamilton Company, CH) equipped with a four 1000 µL channel pipetting arm. Data was collected for 10 min. upon substrate addition on an Infinite M1000 plate-reader (Tecan, CH) at 405 nm using half-area micro-titer plates (Corning, US) with 75 µL assay volume at room temperature (25 °C ± 1). sTF binding studies using S-2288 hydrolysis were performed essentially as described previously^25^, using 0–3 μM sTF and 1 mM S-2288. Kinetic parameters of S-2288 hydrolysis were determined for the FVIIa variants with 0–12.5 mM S-2288, and the *K*_*i*_ for inhibition by pABA was determined in a competitive activity assay using 1 mM S-2288 as described previously^56^. All kinetic and inhibition studies were performed based on the concentration of active protein as determined by the RP-HPLC assay in the absence or presence of 3 μM sTF. Data analysis and curve fitting was conducted using GraphPad Prism 6.0 (GraphPad Software, US).

### Proteolytic activity

Proteolytic activity was measured in a 50 mM Hepes pH 7.4, 100 mM NaCl, 10 mM CaCl_2_, 1 mg/ml BSA, 0.1% PEG8000 buffer with sample preparation and dilution performed on a Microlab STAR Line (Hamilton) equipped with an eight 1000 µL channel pipetting arm. Single point FX activation (15 nM) was measured in the presence of 125 μM PS:PC (70:30 Haematologic technologies, US) using 5 nM activated FVIIa variant in the absence of sTF, and 2.5 pM in the presence of 100 nM sTF. The reaction was allowed to proceed for 30 min. whereafter a quenching buffer (50 mM Hepes pH 7.4, 100 mM NaCl, 80 mM EDTA) was added containing 0.5 mM S-2765 substrate. The hydrolysis of S-2765 was measured on an Infinite M1000 plate-reader (Tecan, CH) at 405 nm using micro-titter plates (Corning, US) with 200 μL assay volume at room temperature (25 °C ± 1). The initial velocity values from the first 5 min. of hydrolysis were derived using linear fitting and normalized to fractions of FVIIa activity.

### Loop building using Rosetta

All 39 variants were generated in silico using the Rosetta^60^ and the next-generation Generalized Kinematic Loop Closure (NGK) protocol^55^ with residues 311{c169}-321{c170I} being rebuild, producing 250 poses for each variant. Two starting templates for FVIIa with TF were used (PDB IDs: 1dan and 4z6a) to sample a wide and narrow angle of the TF-binding helix (306{c164}-311{c169}), respectively. Following loop building, all generated structures were relaxed by restraining the C-alpha to the initial input structure and scoring using the Talaris2014 score function^61–63^. The generated variants were analysed using custom python scripts and PyMOL (Schrödinger LLC, US), followed by statistical analysis and plotting in GraphPad Prism 7.0 (GraphPad Software, US) or with NumPy^64^/matplotlib^65^. All the constructed loops are available for download at Zenodo (https://doi.org/10.5281/zenodo.5513081)^66^.

## Results

### Functional effects of 170 loop truncation in FVIIa

**Figure 2:**
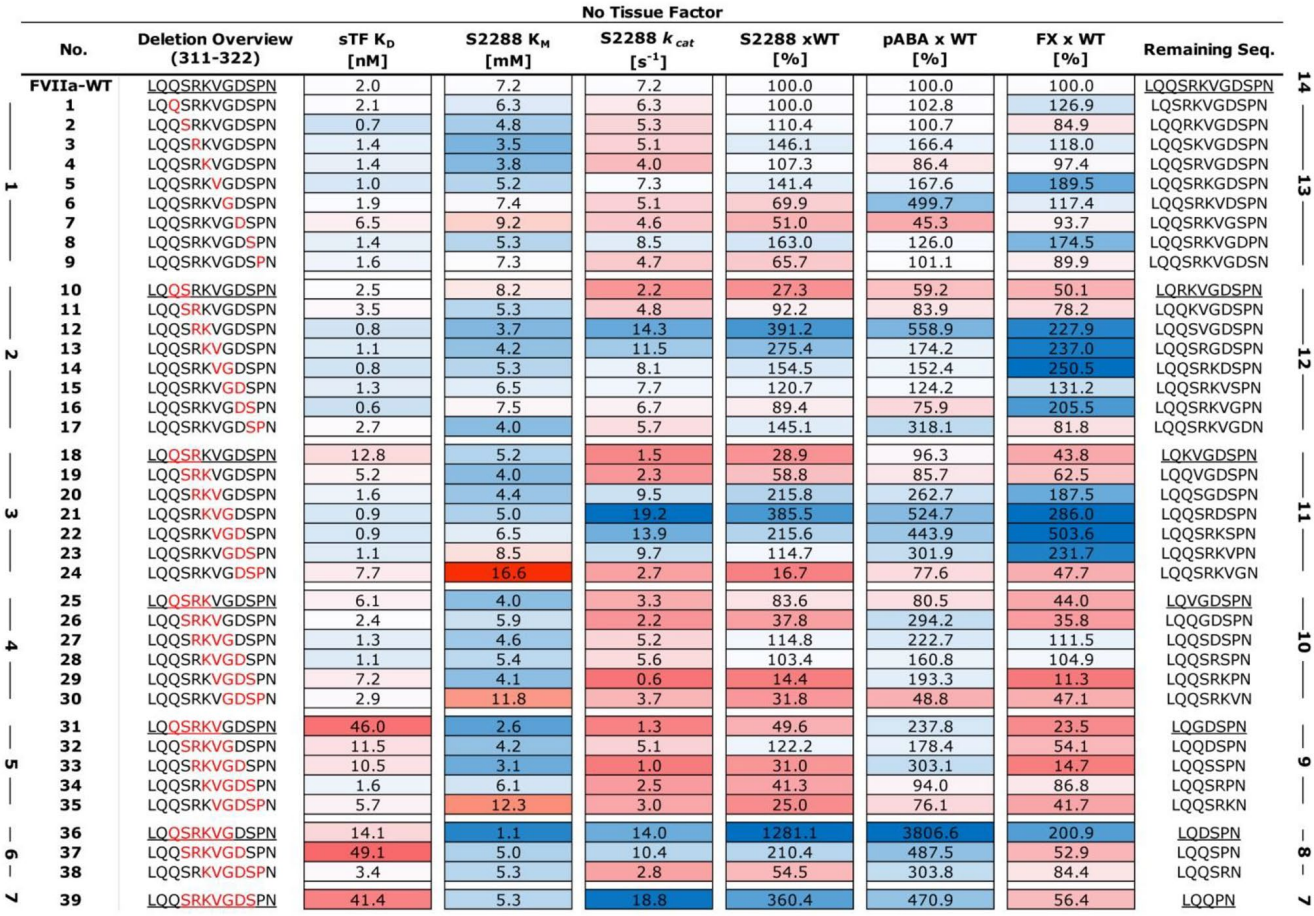
Heatmap of Functional FVIIa Variant Characterization. Soluble tissue factor (sTF) binding as *K*_*d*_ [nM], amidolytic activity as *K*_*M*_ [mM], *k*_*cat*_ [s^−1^] and percentage *k*_*cat*_/*K*_*M*_ compared to FVIIa, pABA inhibition as percentage *K*_*i*_ of FVIIa and Factor X activation as percentage of FVIIa. More active or stronger binding variants are shown in blue and less active or weaker binding variants in red. All data is shown as the mean of duplicate runs without sTF. Variants are sorted by loop length and deletion position (in red) with the start of each sliding window underlined and the resulting loop length listed. (right bold numbering), the number of deleted residues is show on the left.

**Figure 3:**
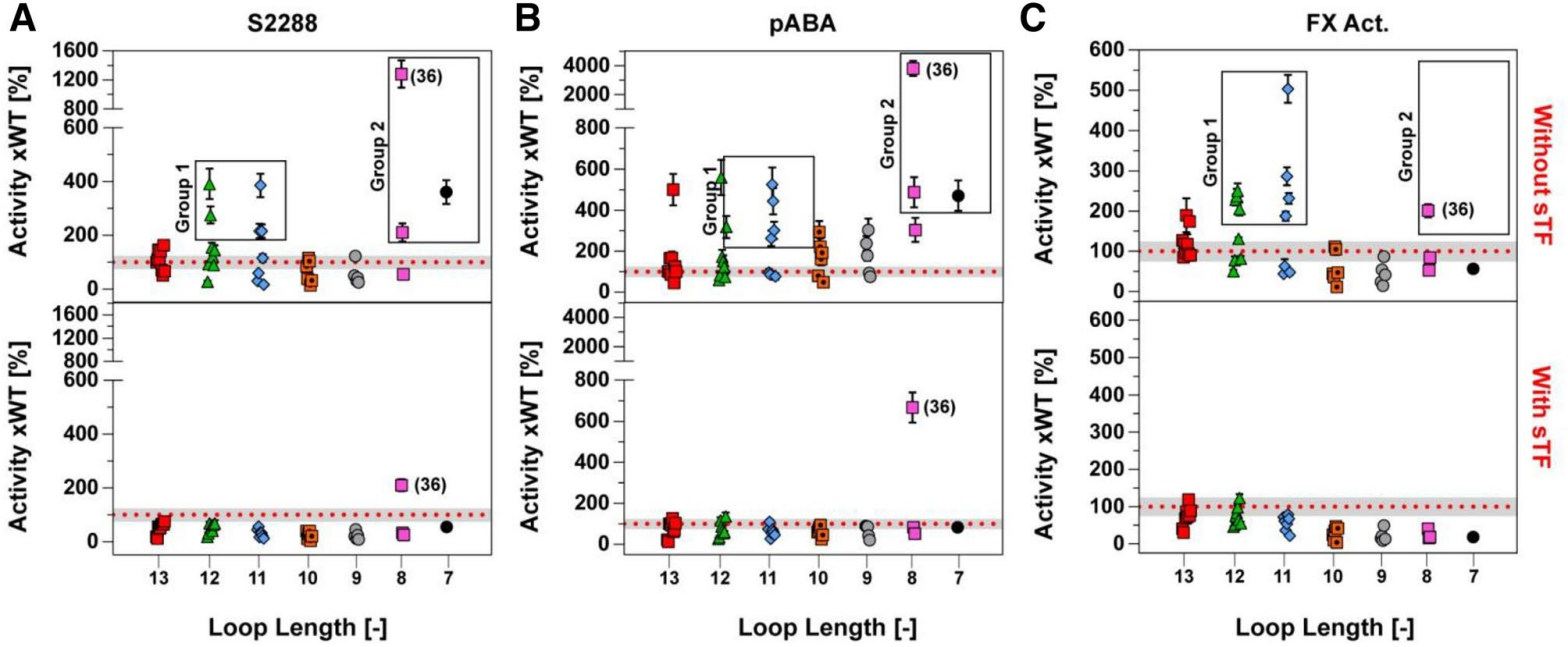
Functional FVIIa Data as a Function of Loop Length Normalized to FVIIa-WT in the absence of sTF (top) or presence of 3 μM sTF (bottom). (**A**) Functional data for hydrolysis of S-2288, (**B**) inhibition of FVIIa variants by pABA and (**C**) Activation of factor X. Group 1 consist of variants no. 12–14 and 20–22 while group 2 contains of variants no. 36, 37 and 39. Data is shown as the mean (n = 2) with high-low error bars for S-2288 and pABA, with mean ± SD (n = 4) for FX activation. The grey shaded area represents one standard deviation for FVIIa-WT around the mean (n = 6) from experiments conducted on different days and different plate positions. Loop length is the remaining number of residues in the 170-loop s for a give variant after a deletion, starting from the wild-type length of 14.

To evaluate the effects of the truncated loops on protease function, we probed the primary specificity pocket (S1), the extended substrate pockets (S2-S4), as well as the full complement of interactions with the natural substrate by performing binding assays using the small-molecule inhibitor pABA, the tri-peptidyl substrate mimic (S-2288), and the full length Factor X, respectively. It is evident from these functional data that both the size of the deletion window and its position affect the activity of a given FVIIa variant in the absence of sTF (Fig. 2). However, any mutational effects are negligible at saturating levels of sTF (supplemental Fig. S2). Indeed, only one variant (no. 36, supplemental Fig. S2) appeared to have an improved amidolytic activity (~ twofold compared to FVIIa-WT) in the presence of sTF, suggesting that the native, extended 170 loop in FVIIa is optimal for the activity of the FVIIa:TF complex. In the absence of sTF, two distinct groups of variants are observed that exhibits both high activity towards S-2288 and a strong pABA binding: The first group consists of variants having a 2–3 residue truncation in the 315{c170C}-319{c170G} positions (no. 12–14 and 20–22, Fig. 2 and 3), while the second group comprises the 6–7 residue truncated variants in the 313{c170A}-320{c170H} positions (no. 36, 37 and 39, Figs. 2 and 3). The most active variant in the absence of sTF (no. 36) was located in the second group and it showed a 12.8-fold increase in amidolytic activity and a 38-fold increased pABA affinity relative to FVIIa. The FX activation of variant no. 36 was, however, only increased twofold, whereas variants found in the first group did show somewhat higher FX activation levels (3–fivefold) when using S-2765 activity as a read-out. Similar findings were found when visualising FX activation by generation of the multichain chain FXa molecule using SDS-PAGE (supplemental Fig. S8).

The deletion of residue P321{c170I} in variants shortened by 3 to 5 residues (no. 24, 30, and 35) had a pronounced negative effect on *K*_*M*_ and, to some extent, on *k*_*cat*_ for S-2288 hydrolysis in the absence of sTF. These effects were also observed as reduced pABA binding and FX activation levels (Fig. 2). Furthermore, deletions in the vicinity of the TF α-helix (residues 311{c169}-LQQS-314{c170B}) was poorly tolerated with a significant effect on the *k*_*cat*_ for hydrolysis of S-2288 for variants no. 10–11, 18–19, 25–26 and 31. In general, loops with 4–5 residue truncation showed poor amidolytic activity, even if the deletion did not include the two regions mentioned above, with the main effect observed on *k*_*cat*_ for these variants (no. 29, 33, and 34). An effect was also observed on the interaction with tissue factor, where variants with shorter loops tended to have a lowered binding affinity in good agreement with previous observations^47^.

### Structure–function relationship of loop shortening

**Figure 4:**
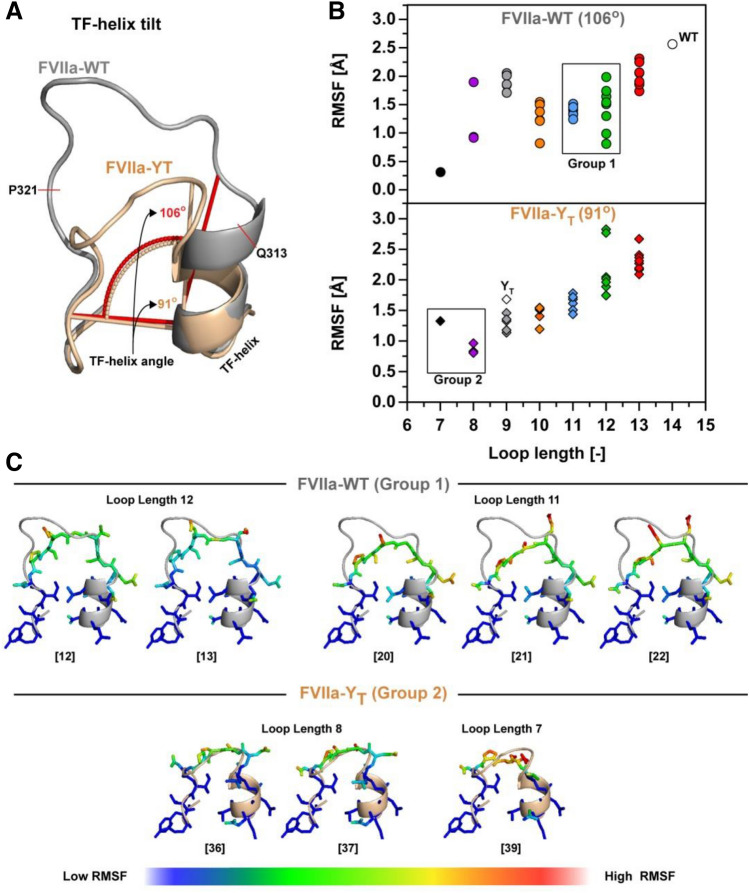
α-helix Angle Determines the Effects of Loop Length on 170 Loop Flexibility. (**A**) Orientation of the TF-helix as defined by the angle between the bottom of the 170 loop and the outer left side of the TF-helix in FVIIa (PDB ID 1dan, grey) and FVIIa-Y_T_ (PDB ID 4z6a, wheat) Q313{c170A} to P321{c170I} was rebuild using the Rosetta NGK protocol. (**B**) Average RMSF for all atoms rebuild in Rosetta with remaining loop lengths colour coded as red (13), green (12), blue (11), orange (10), grey (9), purple (8) and black (7). (**C**) Group 1 and group 2 FVIIa variants build using Rosetta Generalized Kinematic Closure (see supplemental Fig. S3 and S5 for all 39 variants). The root-mean-square fluctuation (RMSF) is defined around the shown geometric mean (N = 250) with low values shown in blue and high values in red.

To investigate the structural effects of 170 loop truncation and to support the functional observations, we built structural models of the 170 loop for all 39 variants using the Rosetta NGK protocol from residue Q313{c170A} to P321{c170I}. Two different starting structures were used to allow for the sampling of a larger conformational space that may better capture longer or short loop variants, with either the wide angle orientation of the TF-interacting α-helix (residues 307{c165}-313{c170A}) from FVIIa-WT^21^ or the more closed orientation determined in a FVIIa variant (FVIIa-Y_T_) induced by the inclusion of the shorter 170 loop from trypsin^25^ (Fig. 4A). The model scores (in Rosetta Energy Units; REU) were calculated in combination with the root-mean square deviation (RMSD) from the lowest scoring structure in order to evaluate clusters of low-energy structures (see supplemental Figs. S4 and S6 for all energy plots). The FVIIa-WT starting structure contained an additional auxiliary gamma-carboxyglutamic domain when compared to FVIIa-Y_T_ and baseline REU levels for the two structures are therefore not identical. Such differences do however not affect the scoring of the lowest energy structures within each generated ensemble. From the modelled structures and the fluctuations around the geometric mean, it was evident that the diversity of structures was dependent on the TF α-helix orientation (Fig. 4B). Loop lengths of 8–9 and 13–14 showed large variation when the structures were built from FVIIa as template, whereas a loop length of 7 or 10–12 in general provided a more restricted ensemble of loop conformations (Fig. 4B). The propensity to generate a narrow ensemble of structures at loop lengths of 11–12 when starting from FVIIa can be interpreted to agree with an increase in functional activity for variants belonging to group 1 (Fig. 4C). Shorter loops of 8–9 that were built on the FVIIa backbone tended to mostly occupy two conformations, one with the loop pointing towards the protease domain and the other pointing into the surrounding solvent (supplemental Fig. S3). Ensembles constructed with the narrower TF α-helix angle of 91° from FVIIa-Y_T_ showed high variability at long loop lengths that decreased as the loop got shorter (Fig. 4B). Stabilization for variants with a loop length of 11–12 was not seen, suggesting that the TF-helix angle in FVIIa allows a certain conformation that stabilizes the loop by favouring interactions with the protease domain. In general, the narrow angle was more compatible with the shorter loop variants (Fig. 4B-C and supplemental Fig. S5) as might be expected based on the tilt angle seen in trypsin (or FIXa and FXa) and FVIIa-Y_T_ (Fig. 1C).

### TF-mimicking interactions facilitate increased functional activity

**Figure 5:**
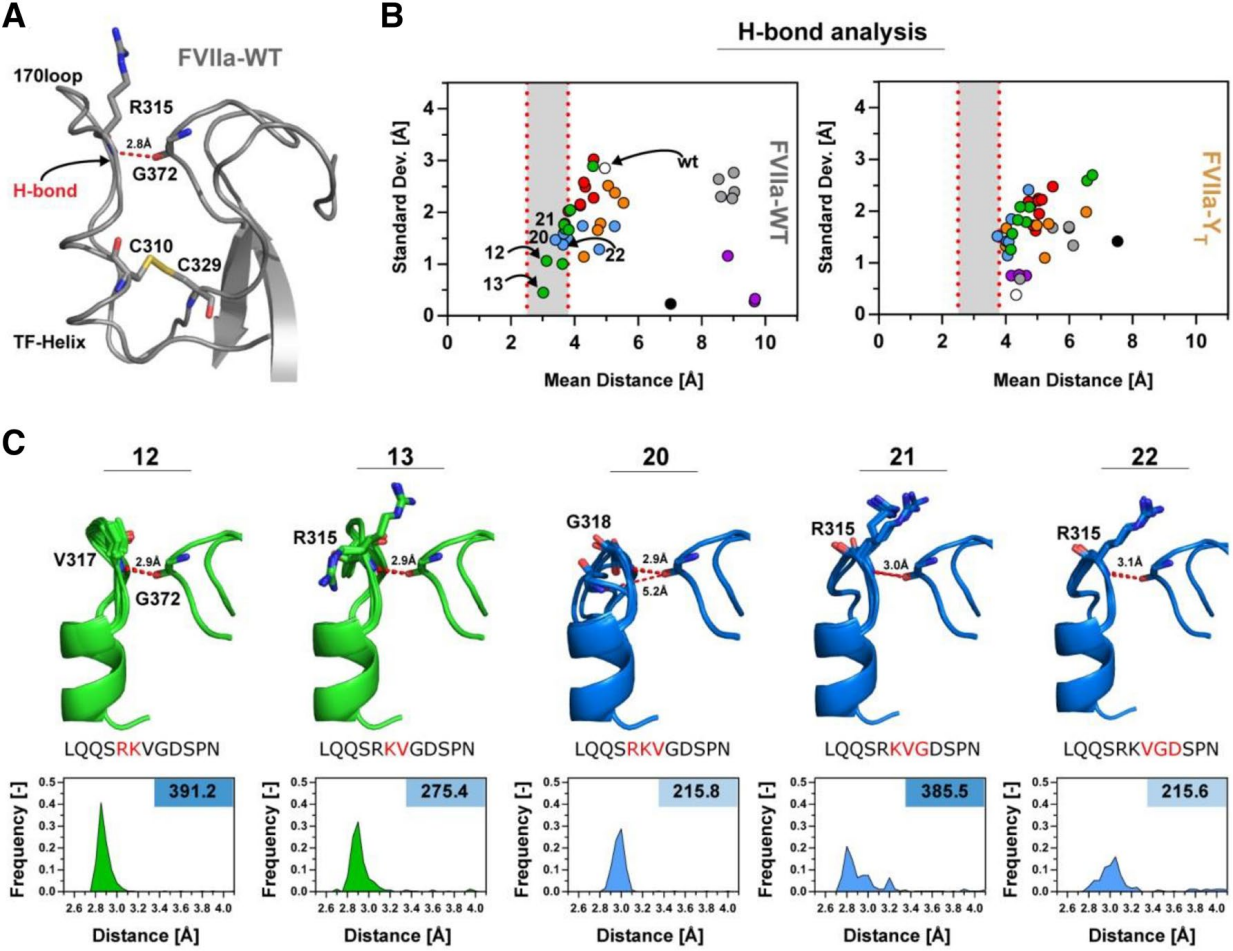
Variants with an Intermediate Loop Length Show TF-Induced-Like Interactions. (**A**) The 170 loop region of FVIIa (PDB ID, 1dan) with the hydrogen bond between R315{c170C} and G372{c223} shown in red, together with the C310{c168}-C329{c182} disulphide-bridge in yellow. (**B**) The mean distance between the residue corresponding to the R315{c170C} backbone nitrogen and G372{c223} oxygen plotted against the standard deviation of the bond length for the FVIIa-WT (left panel) or FVIIa-Y_T_ backbone (right panel). Bond lengths of 2.5–3.8 Å are marked in grey for loop conformations with possible stable hydrogen bonds including variants 12–13 and 20–22. (**C**) Overview of group 1 variants with high activity including 10 modelled structures with the lowest Rosetta Energy (see supplemental Fig. S4) with the average hydrogen-bond distance shown in red. Frequency histograms for variants with distances in the 2.5–4.0 Å range showing a favourable distribution for loop lengths 11–12. The corresponding S-2288 activity relative to FVIIa in the right top-corner and deleted amino acids are coloured in red on the shown sequence.

We investigated the variants from group 1 in more detail to shed light on the structural features underpinning the reduced sample space of loop conformation and its relation to the observed increased activity. Previous studies of FVIIa emphasized that a hydrogen bond located at the top of the 170 loop, between the backbone of R315{c170C} and G372{c223} (Fig. 5A), is involved in TF mediated activity enhancement^67^. By measuring the distance between the backbone nitrogen of R315{c170C} (or the residue in its place) and the G372{c223} carbonyl oxygen for the variants, we found that a loop length of 11–12 results in a loop conformation with propensity for the correct orientation for hydrogen-bond formation (Fig. 5B). This effect was not observed in a control setup where the 14 amino acids of the wild type molecule were rebuilt. Interestingly, an increased propensity to sample an electrostatically favourable R315{c170C}-G372{c223} interaction was associated with increased activity for the variants in group 1 (Fig. 5C). Plots of model energy (in REU) against RMSD for the variants in this group (12–13, 20–22) showed a funnel-like shape (supplemental Fig. S4), consistent with the presence of an ensemble of energetically favourable structures for each variant (the 10 best scoring structures are depicted on Fig. 5C). Interestingly, this favourable interaction was not observed when constructing the loops based on the FVIIa-Y_T_ structure, suggesting that the ~ 15° change in TF-helix orientation is unfavourable for this loop length (Fig. 5B).

### Trypsin-like interactions between the 170 loop and protease domain confers activity

**Figure 6:**
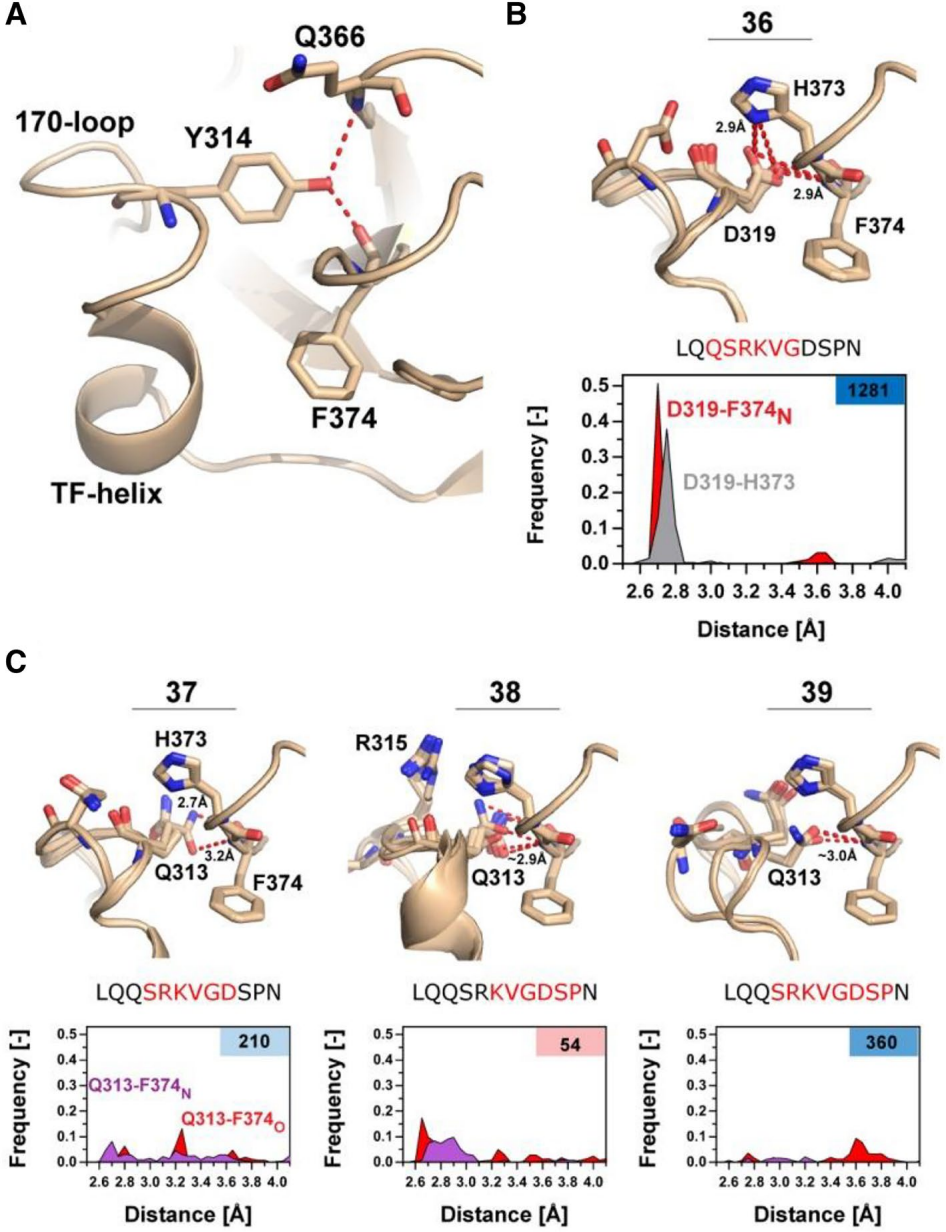
Shorter Loop Lengths Allow for Trypsin-like Interactions with the Protease Domain. (**A**) Overview of trypsin-like interaction in a FVIIa-trypsin variant (PDB ID: 4z6a), with Y314 (172c) inserting into the protease domain, stabilizing Q366{c217} and F374{c225} by polar contacts (red) as observed in wild-type trypsin. (**B**) 10 models with lowest RMSD/REU of the most active variant (no 36.) from group 2, reconstructed using the NGK protocol. D319{c170G} makes polar contacts (red) with the backbone nitrogen of F374{c225} and sidechain of H373{c224} to stabilize the S1-pocket. A frequency histogram of the polar contacts between D319{c170G}-H373{c224} (grey) or D319-F374_N_ (red) highlights the favourability of the interaction (n = 250) with S-2288 activity relative to FVIIa in the right top-corner and deleted amino acids are coloured in red on the shown sequence. (**C**) Overview of variants 37–39 with 3 groups of 5 modelled structures selected from RMSD groupings showing the lowest REU (see supplemental Fig. S6). Average hydrogen-bond distance is shown in red when the distance is < 3.5 Å. Frequency histograms (n = 250) of the polar contacts between Q313{c170A} -F374_N_{c225} (red) or Q313{c170A}-F374_O_{c225} (purple).

A more detailed look at the computational models generated for variants with a loop length of 7–8 belonging to group 2 revealed that interactions between the 170 loop and the protease domain are analogous to those found in wild-type trypsin and FVIIa-Y_T_ (Fig. 6A-C). In variant no. 36 with the highest amidolytic activity, D319{c170G} is inserted into a pocket between Q366{c217} and F374{c225} in many modelled structures to form polar contacts with the backbone nitrogen of F374{c225} and an imidazole nitrogen of H373{c224} (Fig. 6B). This is in agreement with the significant positive effect on *K*_*M*_ for S-2288 hydrolysis (1.1 mM) when compared with FVIIa (7.2 mM) resulting in an overall 12.8-fold increase in activity when compared to FVIIa-WT. Only variant (no. 31) has D319{c170G} in the same position and it appears to exhibit a similar stabilizing effect, albeit to a lesser degree. This variant also showed an improved *K*_*M*_ (2.6 mM) as would be expected, but a significantly compromised *k*_*cat*_. For the remaining seven variants (33–35 and 37–39) with a similar loop length, D319{c170G} is replaced with Q313{c170A}, resulting in a changed polar-contact pattern. In those variants, Q313{c170A} form polar contacts with either the backbone nitrogen or carbonyl of F374{c225}, but not both for variants 37–39 (Fig. 6C). This observation agrees with a modest improved S-2288 activity for all variants, except variant 38 that shows a reduced *k*_*cat*_. These observations align well with the funnel-like shape of model energy (in REU) against RMSD plots (supplemental Fig. S6) with 15 energetically favourable models shown on Fig. 6B-C.

## Discussion

We applied computational modelling combined with a systematic loop truncation approach with a sliding window size of 1–7 deletions, that in combination with functional studies, provides a powerful method for detailed structure–function elucidation of surface-exposed loops in trypsin-like protease. These results will complement structural studies using X-ray crystallography or cryo-EM but can also be a viable substitute when these are unavailable or co-factors/inhibitors that may have a distorting effect on surface exposed loops have to be employed to achieve high quality crystals. In addition, the extended space of considered truncations employed here allows for the detection of variants that would be ignored in approaches relying on random/direct mutagenesis, where 2–3 residues are truncated.

Using the sliding deletion window, 39 protease variants were generated. The functional characterization showed that high-activity variants tended to cluster in two groups with striking similarities to previous findings. The first group comprised loop lengths of 11–12 (2–3 residue deletions) when deleting outside the residues forming part of the TF α-helix (307{c165}-312{c170}) (Fig. 3). The structural models generated using the NGK approach with FVIIa as a template suggested a structural stabilization for these variants similar to that reported to be induced by TF binding^67^, and favouring loops that had distances between R315{c170C} and G372{c223} that could accommodate favourable electrostatic interactions (Fig. 5B). This recapitulation of previous findings demonstrates the applicability of the method for examining the function of surface-exposed loops in TLPs and supports the notion of evolutionary truncation/variability to achieve specific functions in TLPs. If, however, a FVIIa variant with 170 loop from trypsin (FVIIa-Y_T_) with a narrower TF α-helix angle was used as a template, the productive electrostatic interaction between R315{c170C} and G372{c223} was not favoured. Likely, this segment of the wild-type FVIIa is responsive to TF-mediated allosteric enhancement activity by stabilizing the S1-pocket^16,31^, which is in good agreement with the increased functional activity observed here. Interestingly, variants with only a single deletion seemed to have a loop length that results in a high number of possible conformations for the reconstructed loops, consistent with a small loss or no change in activity. Taken together, these data support the notion that the full length 170 loop of FVIIa is too flexible to enable the R315{c170C}**-**G372{c223} hydrogen bond interaction that appears important for protease activity and that TF binding to FVIIa may tether the loop down and thereby stabilizing R315{c170C} for a productive interaction with G372{c223}^68,69^. Another observation supporting this interpretation was that only a single variant (no. 36) had a higher amidolytic activity in the presence of sTF than FVIIa:sTF, suggesting that the extended 170 loop with a length of 14 residues is tailored for TF-mediated increased FVIIa activity.

The second group consisted of variants with 6–7 residue truncations, which included the most active variant in the library carrying a 12.8**-**fold increased amidolytic activity (no. 36). It was evident from the reconstructed loop models (using FVIIa-Y_T_ as a template) that the six-residue deletion in variant 36 moved residue D319{c170G} into a position to interact with the body of the protease domain. D319{c170G} appears to likewise stabilize the S1-pocket through interactions with activation-loop 3 at the backbone nitrogen of F374{c225} and the sidechain of H373{c224}. Surprisingly, this interaction is congruent to that observed for Y172c (Supplemental Fig S7) in trypsin^70^ and a FVIIa variant with the 170 loop grafted from trypsin^25,47,71^, supporting the notion that our approach can be used to identify “hot-spots” for further engineering. In addition, a number of other variants with similar loop lengths showed a corresponding placement and orientation of residue Q313{c170A}, but the frequency of occupying the stable conformation was much lower than that observed for variant no. 36. This is cause by the single hydrogen bond acceptor nature of Glutamine that only allows for production interaction with either F374{c225} or H373{c224}, correlating well with the modest increase in amidolytic activity. None of these interactions were observed when FVIIa-WT was used as a template for the loop reconstruction as the wider angle of the TF-helix shifted both D319{c170G} and Q313{c170A} out-of-range of where they could interact productively with the protease domain. It was furthermore observed that variants with suggested high propensity to form polar contacts seemed to exhibit a notable negative effect on sTF binding affinity (Fig. 2), in good agreement with previous observations for a FVIIa variant with the 170 loop from trypsin and corresponding tilt of the TF α-helix^25,47,71^.

The narrow helix angle induced in FVIIa-Y_T_ and variants generated here are in good agreement with more intrinsically active proteases such as FXa or thrombin, where the 170 loop is truncated by 5 residues. In these proteases a structural water network, supported by residues from the 170 loop and the change in helix angle, is likely to be a foundation for the high intrinsic activity^25,71–73^. The helix angle is even further narrowed in wild-type trypsin, allowing optimal insertion of Y172c to stabilize the S1 pocket and confer constitutive activity to the protease^5,70^. Thus, for a general applicability toward TLPs where representative loop and loop-anchor conformations spanning the functional spectrum are not available, it may be necessary to sample a continuum of helix angles upon which computational loop reconstruction is performed.

Using the systematic deletion approach presented here, we were able to capture many of the important structural features of the 170 loop reported over the last several decades of work on FVIIa. In addition, it was revealed that the variant with highest activity was achieved by a six-amino acid truncation with a deletion-window shifted 3 residues towards the N-terminus when compared to the shorten loop of ancestral trypsin. Most likely, such a variant could not have been detected with the sampling space of traditional directed/random mutagenesis. Taken together these findings suggest that the methodology is useful as a screening tool for identifying “hot-spots” from a wide initial pool of *in-silico* designed variants that will significantly reduce the amount of experimental testing required to engineer highly active protease variants. The presented approach furthermore highlights the diversity afforded TLPs, all building on a remarkably similar beta-sandwich structure (“The Trypsin fold”) that combined with highly variable surface exposed loops, enables this class of vital enzymes to span the evolutionary need of constitutively active proteases (e.g., Trypsin and variant 36) to that of highly co-factor control homeostatic systems (e.g., FVIIa-WT with tissue factor).

## Supplementary Information

Supplementary Information.
