# Supplementary material for: A systematic approach for evaluating the role of surface-exposed loops in trypsin-like serine proteases applied to the 170 loop in coagulation factor VIIa
Source: Sci Rep. 2022 Mar 8;12:3747. doi: 10.1038/s41598-022-07620-7 (PMC8904457; doi:10.1038/s41598-022-07620-7)
Supplement: Supplementary file 1 — Supplementary Information. [file 41598_2022_7620_MOESM1_ESM.pdf]

Supplemental information for

A Systematic Approach for Evaluating the Role of Surface-Exposed Loops in Trypsin-like Serine Proteases Applied to the 170 loop in Coagulation Factor VIIa

Anders B. Sorensen,[1,2] Per Jr. Greisen,[1] Jesper J. Madsen,[3,4] Jacob Lund,[1] Gorm Andersen,[1] Pernille G. Wulff-Larsen,[1] Anette A. Pedersen,[1] Prafull S. Gandhi,[1] Michael T. Overgaard,[2] Henrik Østergaard,[1] Ole H. Olsen[1,5,*]

[1] Global Research, Novo Nordisk A/S, 2760 Måløv, Denmark

[2] Department of Chemistry and Bioscience, Aalborg University, 9220 Aalborg, Denmark

[3] Global and Planetary Health, College of Public Health, University of South Florida, Tampa, FL 33612, USA

[4] Department of Molecular Medicine, Morsani College of Medicine, University of South Florida, Tampa, FL 33612, USA

[5] Current affiliation: Novo Nordisk Foundation Center for Basic Metabolic Research, Section for Metabolic Receptology, University of Copenhagen, Blegdamsvej 3b, DK-2200 Copenhagen, Denmark

Supplemental methods

FX Activation Kinetics

500 nM of individual FVIIa-variants were pre-incubated with 20 µM PS:PC (25:75 Haematologic technologies, US) in 50 mM HEPES pH 7.4, 100 mM NaCl, 10 mM CaCl2, 0.1 % PEG8000 for 5 minutes at room temperature. 500 nM FVIIA-WT in the absence and presence of 100 nM soluble tissue factor (sTF) where used for comparison. Plasma-derived FX (Haematologic technologies, US) was then added to a final concentration of 2.5 µM. At indicated time points, a small portion of the sample was withdrawn, mix with NuPAGE™ LDS Sample Buffer and NuPAGE™ Sample Reducing Agent (Thermo Fisher Scientific, US) and heated at 70 °C for 10 minutes. Samples were separated in 4-12% Bis-Tris SDS-PAGE (Thermo Fisher Scientific, US) and stained with coomassie brilliant blue. Plasma-derived FXa (Haematologic technologies, US) were added as a control. Gel band intensities (area under the curve) were evaluated by an in-house dedicated python script, using the FXa HCβ band to determine initial FX-activation rates using a first order equation in GraphPad Prism.

This supplementary document contains the following figures:

Figure S1 Sequence alignment of the catalytic domain of selected trypsin-like proteases

Figure S2 Heatmap of functional FVIIa variant characterization with 3 µM sTF

Figure S3: RMSF plots of all reconstructed loops from FVIIa-WT (1dan)

Figure S4: REU vs RMSD scatterplots for FVIIa-WT (1dan)

Figure S5: RMSF plots of all reconstructed loops from FVIIa-Y_T (4z6a)

Figure S6: REU vs RMSD scatterplots for FVIIa-Y_T (4z6a)

Figure S7: Comparison of trypsin and FVIIa var. 36

Figure S8: FX-activation kinetics of selected FVIIa-variants visualized by SDS-PAGE

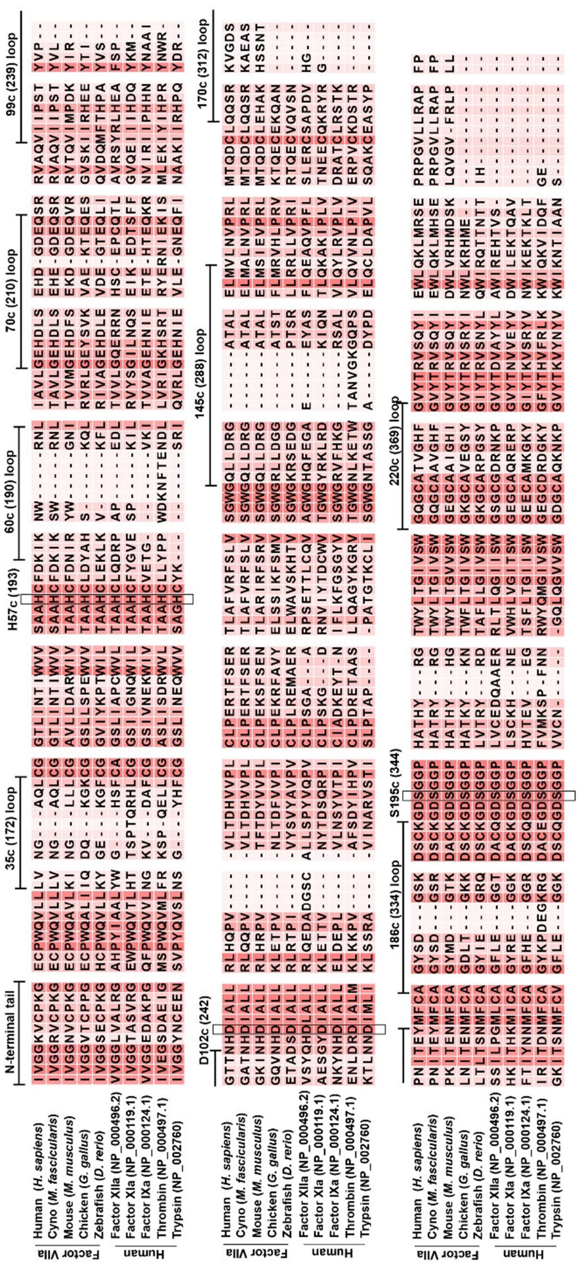

Supplemental Figure S1. Sequence alignment of the catalytic domain of selected trypsin-like proteases using the PROMALS3D webserver. Consensus numbering based on Chymotrypsin has been used to mark surface-exposed loops with extended loop regions denoted with capital letters. FVII numbering is shown in parentheses for reference. The red colour gradient indicates conservation level with intense colour showing the highest level of conservation.

3 uM Tissue Factor

	No.	Deletion Overview	S2288 K_M [mM]	S2288 k_{cat} [s^{-1}]	S2288 xWT [%]	pABA x WT [%]	FX x WT [%]	Remaining Seq.	
	FVIIa-WT	LQQSRKVGDSPN	1.1	36.1	100.0	100.0	100.0	LQQSRKVGDSPN	14
	1	LQQSRKVGDSPN	3.7	20.3	17.3	22.5	41.1	LQSRKVGDSPN	
	2	LQQSRKVGDSPN	5.7	19.7	10.9	13.8	30.7	LQQRKVGDSPN	
	3	LQQSRKVGDSPN	1.7	29.5	55.0	100.8	0.0	LQQSKVGDSPN	
	4	LQQSRKVGDSPN	1.8	27.2	48.1	101.7	67.8	LQQSRVGDSPN	
1	5	LQQSRKVGDSPN	1.7	26.5	50.4	94.4	86.3	LQQSRKGDSPN	13
	6	LQQSRKVGDSPN	1.3	26.3	63.3	127.8	72.9	LQQSRKVDSPN	
	7	LQQSRKVGDSPN	2.3	48.2	65.3	61.9	118.8	LQQSRKVGSPN	
	8	LQQSRKVGDSPN	1.6	32.9	64.0	69.3	76.0	LQQSRKVGDPN	
	9	LQQSRKVGDSPN	1.7	39.1	74.1	104.0	88.8	LQQSRKVGDSN	
	10	LQQSRKVGDSPN	3.1	17.8	18.0	27.2	45.6	LQRKVGDSPN	
	11	LQQSRKVGDSPN	2.7	25.7	30.1	35.0	52.1	LQQKVGDSPN	
	12	LQQSRKVGDSPN	1.6	34.0	67.5	117.9	74.9	LQQSVGDSPN	
	13	LQQSRKVGDSPN	2.0	33.4	52.2	88.0	84.0	LQQSRGDSPN	
2	14	LQQSRKVGDSPN	1.7	27.2	50.5	70.2	98.4	LQQSRKDSPN	12
	15	LQQSRKVGDSPN	2.8	34.8	39.5	55.1	64.6	LQQSRKVSPN	
	16	LQQSRKVGDSPN	1.6	32.3	63.0	60.1	123.5	LQQSRKVGPN	
	17	LQQSRKVGDSPN	1.6	33.6	66.9	136.0	55.4	LQQSRKVGDN	
	18	LQQSRKVGDSPN	1.8	24.6	43.8	74.0	72.0	LQKVGDSPN	
	19	LQQSRKVGDSPN	1.5	26.3	56.0	109.1	61.1	LQQVGDSPN	
	20	LQQSRKVGDSPN	5.6	27.7	15.6	27.9	36.0	LQQSGDSPN	
3	21	LQQSRKVGDSPN	3.3	35.9	34.6	55.2	53.9	LQQSRDSPN	11
	22	LQQSRKVGDSPN	4.6	45.3	31.2	70.3	78.7	LQQSRKSPN	
	23	LQQSRKVGDSPN	4.2	27.9	21.1	59.6	66.9	LQQSRKVPN	
	24	LQQSRKVGDSPN	4.4	14.9	10.9	46.1	21.5	LQQSRKVGN	
	25	LQQSRKVGDSPN	2.6	31.9	38.9	60.5	24.6	LQVGDSPN	
	26	LQQSRKVGDSPN	2.6	8.9	10.7	68.5	10.2	LQQGDSPN	
4	27	LQQSRKVGDSPN	2.7	24.7	29.1	83.9	35.3	LQQSDSPN	10
	28	LQQSRKVGDSPN	2.2	26.3	38.6	94.5	46.5	LQQSRSPN	
	29	LQQSRKVGDSPN	5.0	4.5	2.8	26.2	3.4	LQQSRKPN	
	30	LQQSRKVGDSPN	5.3	31.9	19.2	45.8	41.7	LQQSRKVN	
	31	LQQSRKVGDSPN	1.4	8.9	19.5	90.3	14.8	LQGDSPN	
	32	LQQSRKVGDSPN	1.9	27.2	44.5	85.7	21.1	LQQDSPN	
5	33	LQQSRKVGDSPN	2.1	6.6	9.8	87.1	8.9	LQQSSPN	9
	34	LQQSRKVGDSPN	2.3	16.3	23.0	43.4	49.0	LQQSRPN	
	35	LQQSRKVGDSPN	8.7	19.9	7.3	21.3	13.1	LQQSRKN	
	36	LQQSRKVGDSPN	0.2	15.6	210.0	666.8	41.0	LQDSPN	
6	37	LQQSRKVGDSPN	2.5	24.9	32.2	83.9	15.5	LQQSPN	8
	38	LQQSRKVGDSPN	2.0	15.2	24.3	52.4	18.0	LQQSRN	
7	39	LQQSRKVGDSPN	2.5	42.6	54.2	82.8	18.3	LQQPN	7

Supplemental Figure S2. Heatmap of Functional FVIIa Variant Characterization With 3 μM Soluble Tissue Factor**.** Amidolytic activity as K_M [mM], k_{cat} [s^{-1}] and percentage k_{cat}/K_M compared to FVIIa, pABA inhibtion as percentage K_i of FVIIa and Factor X activation as percentage of FVIIa. More active or stronger binding variants are shown in blue and less active or weaker binding variants in red. All data is shown as the mean of duplicate runs with 3 μM soluble tissue factor. Variants are sorted by loop length and deletion position (in red) with the start of each sliding window underlined and the resulting loop length listed.

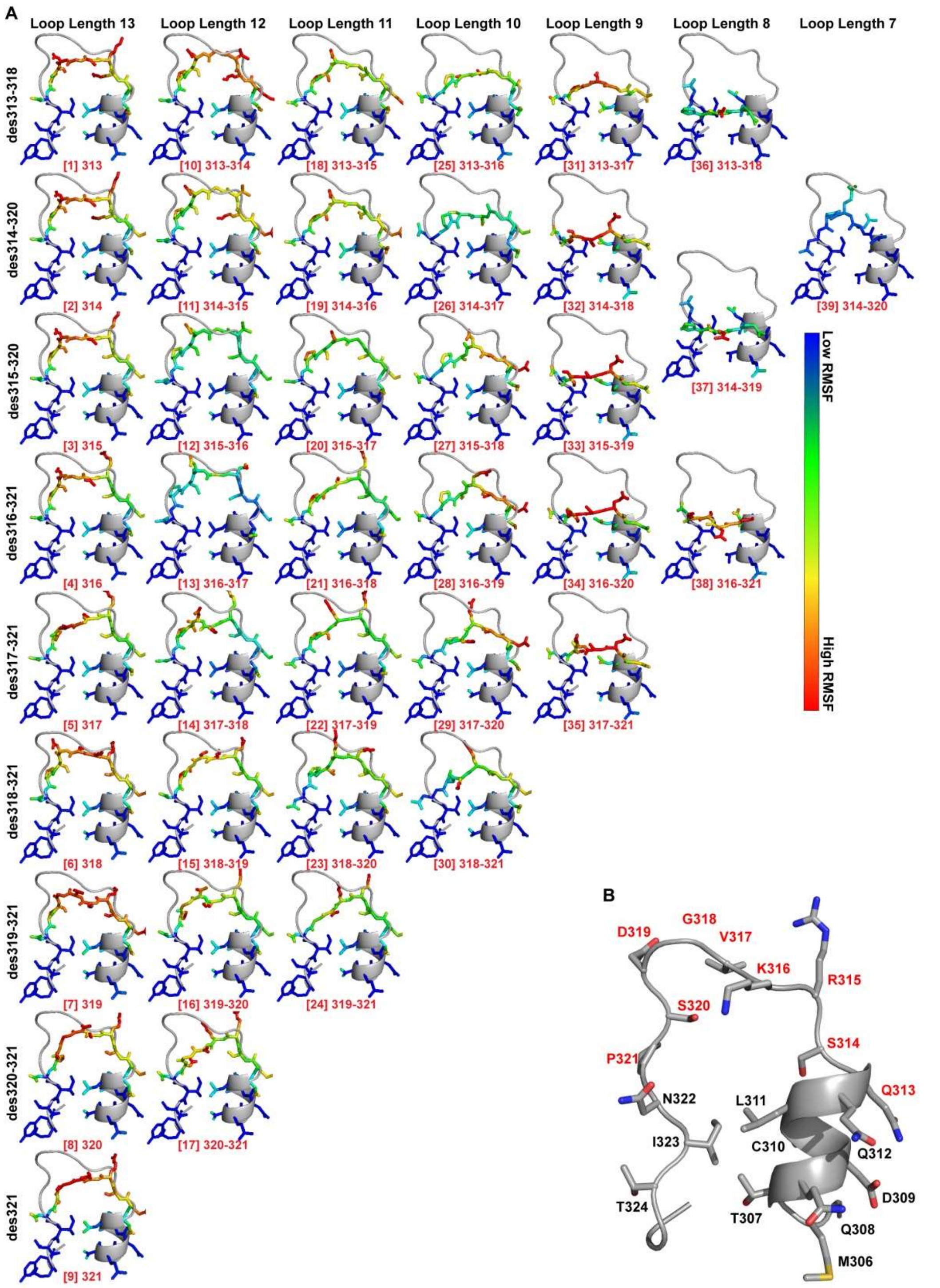

Supplemental Figure S3. (A) Overview of the reconstructed 170-loop for all 39 variants ordered according to loop length and deletion position using FVIIa-WT (PDB ID: 1dan) as a template. High variation in the position traced by the Rosetta NGK protocol is shown in red, low is shown in blue. **(B)** Only positions 311{c169}-321{c170I} was rebuild using the protocol as shown on the template structure of FVIIa-WT.

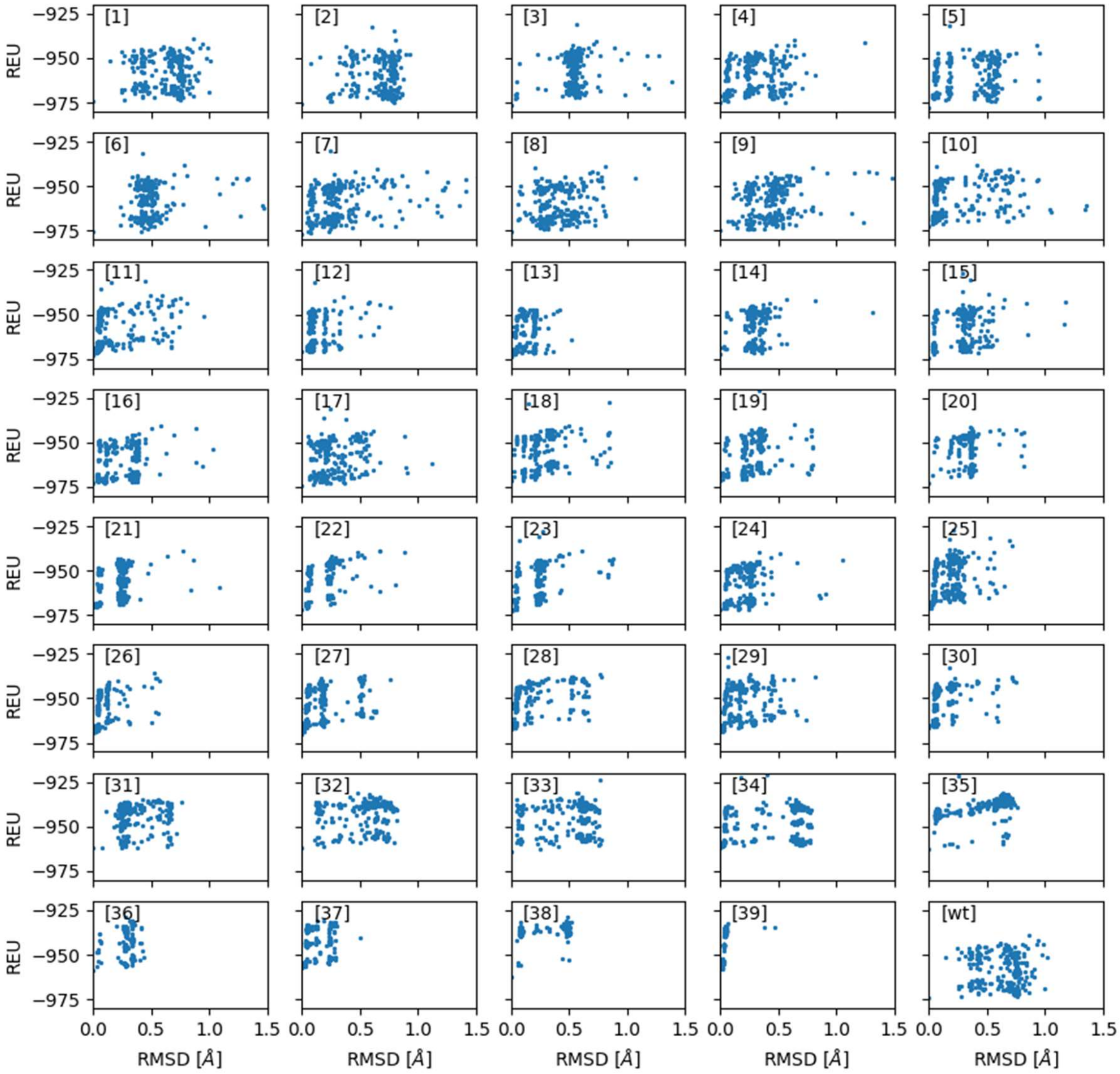

Supplemental Figure S4. Overview of model energy (in Rosetta Energy Units; REU) calculated using the Talaris2014 energy function plotted against root-mean-square deviation for all 39 generated variants with the FVIIa-WT structure (PDB ID: 1dan) as a template.

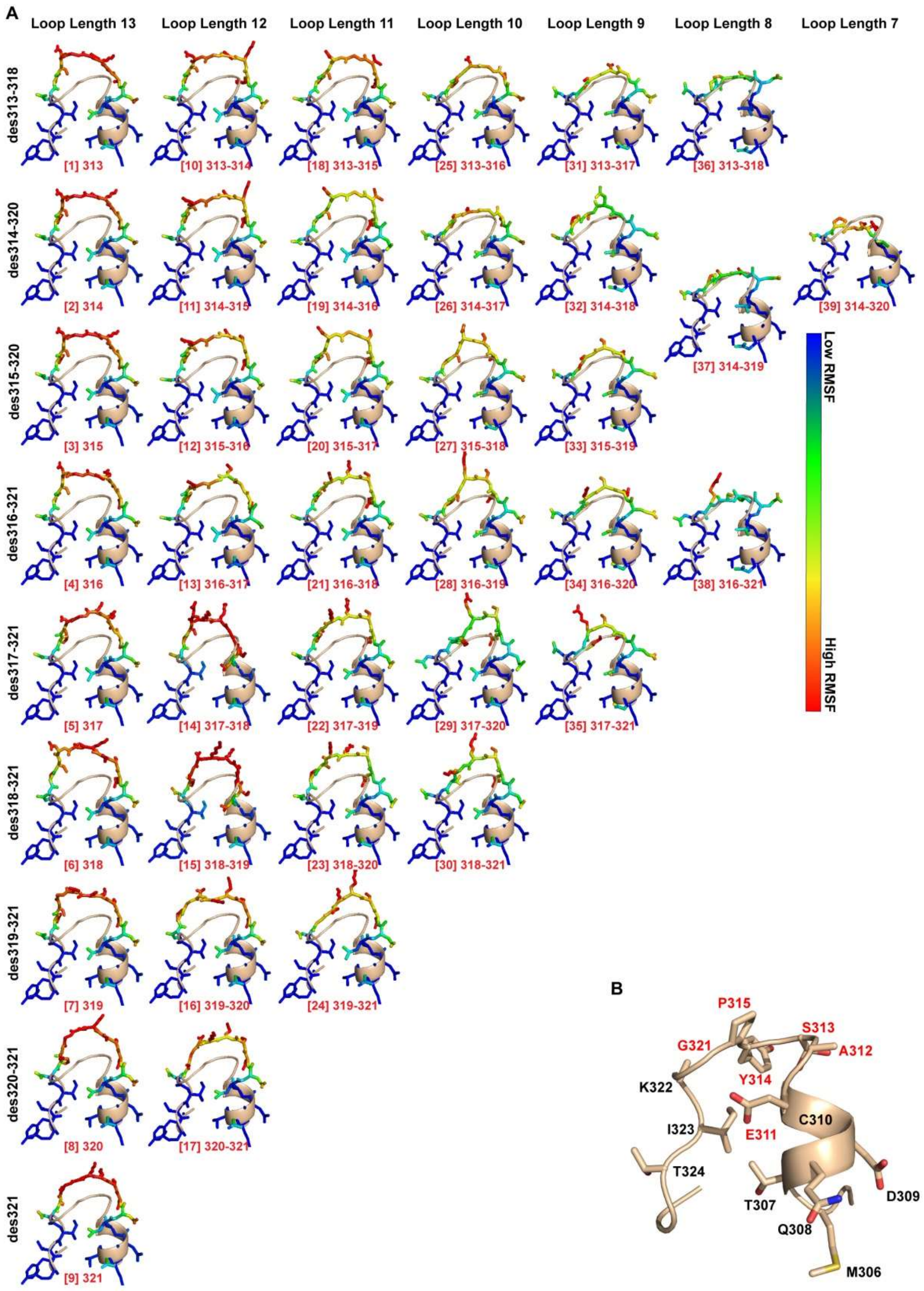

Supplemental Figure S5. (A) Overview of the reconstructed 170-loop for all 39 variants ordered according to loop length and deletion position using FVIIa-Y_T (PDB ID: 4z6a) as a template. High variation in the position traced by the Rosetta NGK protocol is shown in red, low is shown in blue. **(B)** Only position 311{c169}-321{c170I} was rebuild using the protocol as shown on the template structure of FVIIa-Y_T.

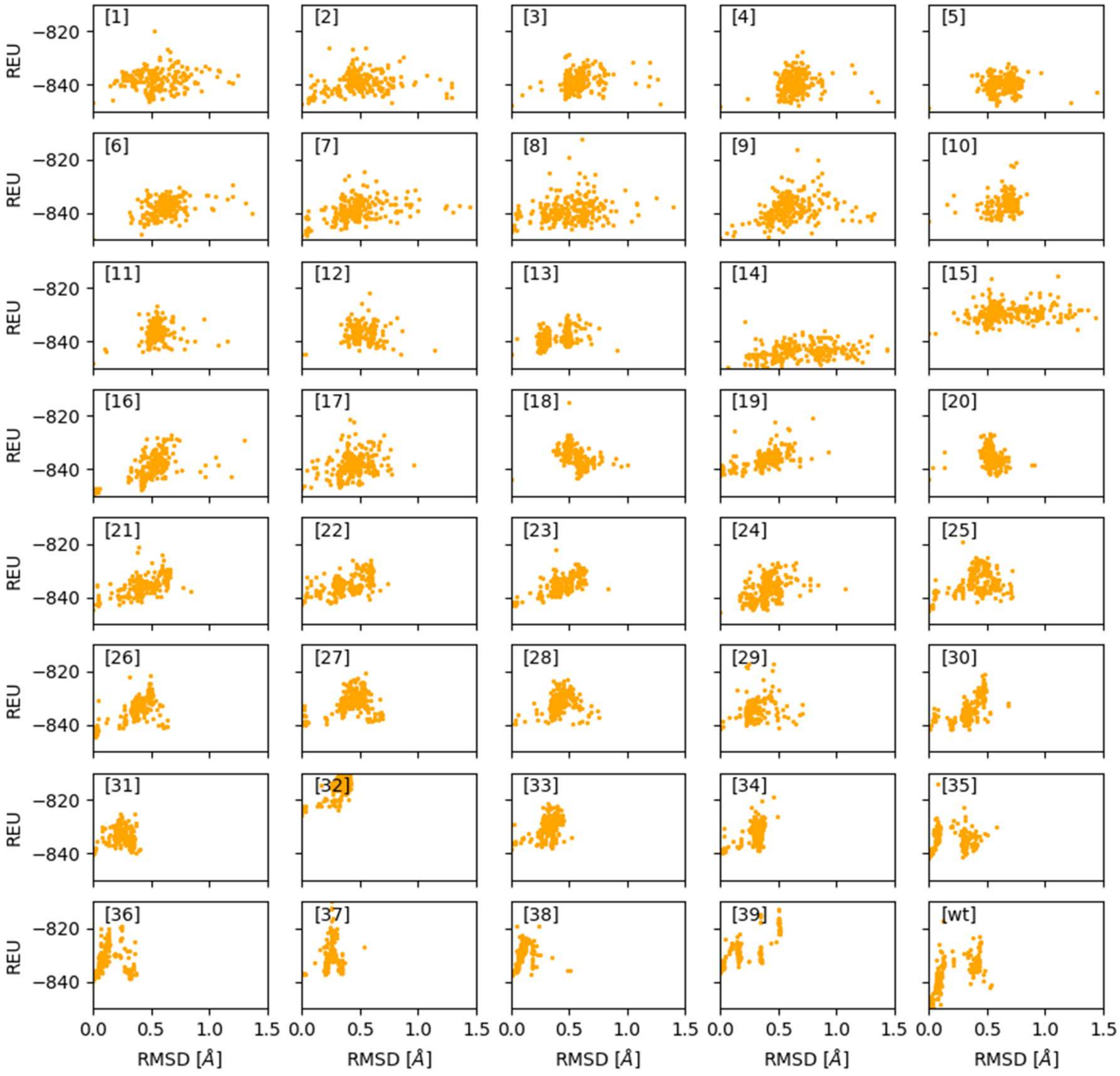

Supplemental Figure S6. Overview of model energy (in Rosetta Energy Units; REU) calculated using the Talaris2014 energy function plotted against root-mean-square deviation for all 39 generated variants with the FVIIa-Y_T structure (PDB ID: 4z6a) as a template.

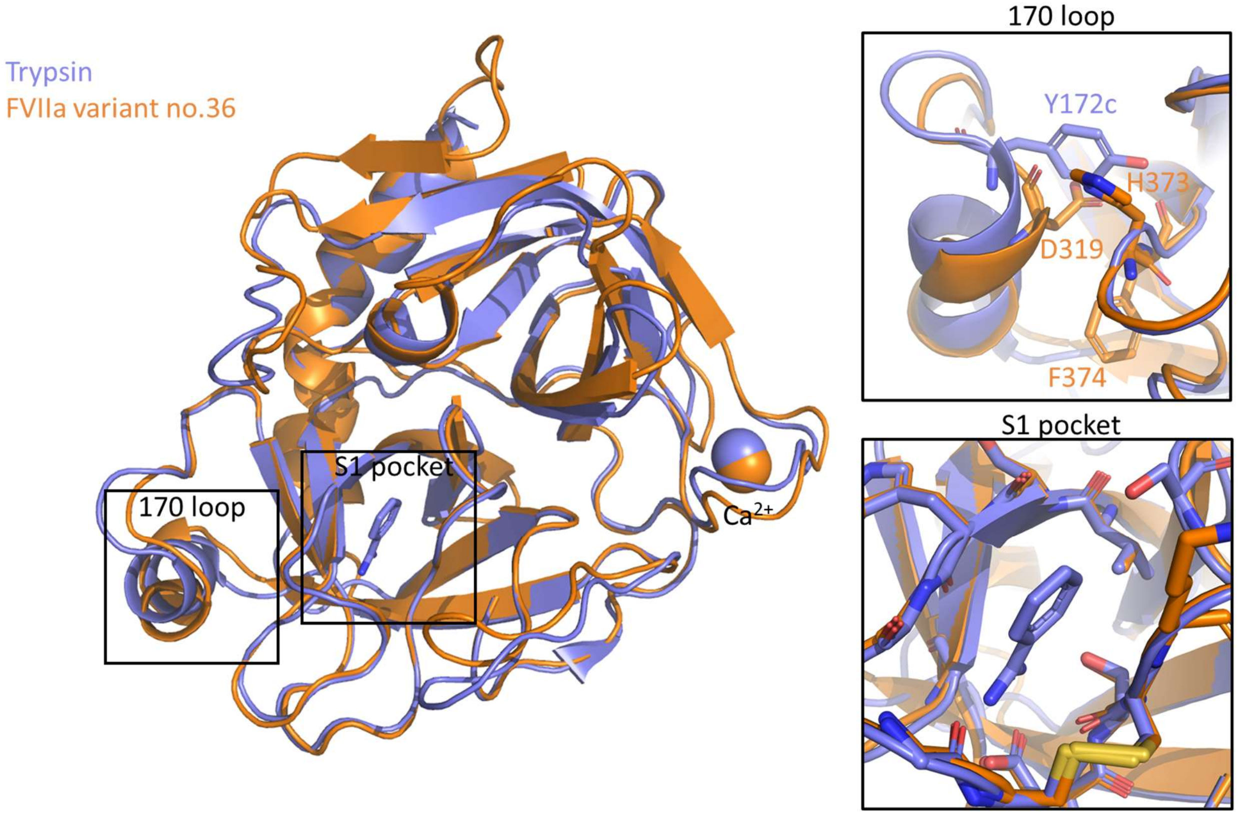

Supplemental Figure S7. Comparison of crystal structures for wild type trypsin (PDB ID: 1j8a) and FVIIa variant no. 36 build on FVIIa PDB ID 4z6a. Inserts show zoomed views of the 170 loop and the S1 substrate pocket, respectively.

A

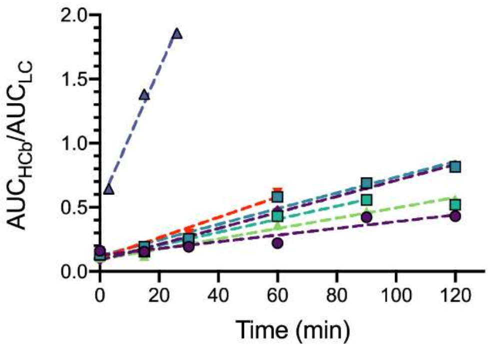

	FX-activation gel densitometry Initial rate (min⁻¹)	FX-activation gel densitometry FX x WT	S-2765 FX Activation FX x WT
variant12	0.0061	234	227.9
variant13	0.0051	196	237.0
variant20	0.0040	154	187.5
variant22	0.0079	302	503.6
variant36	0.0062	238	200.9
wt	0.0026	100	100.0
wt/sTF	0.0528	2017	N/A

B

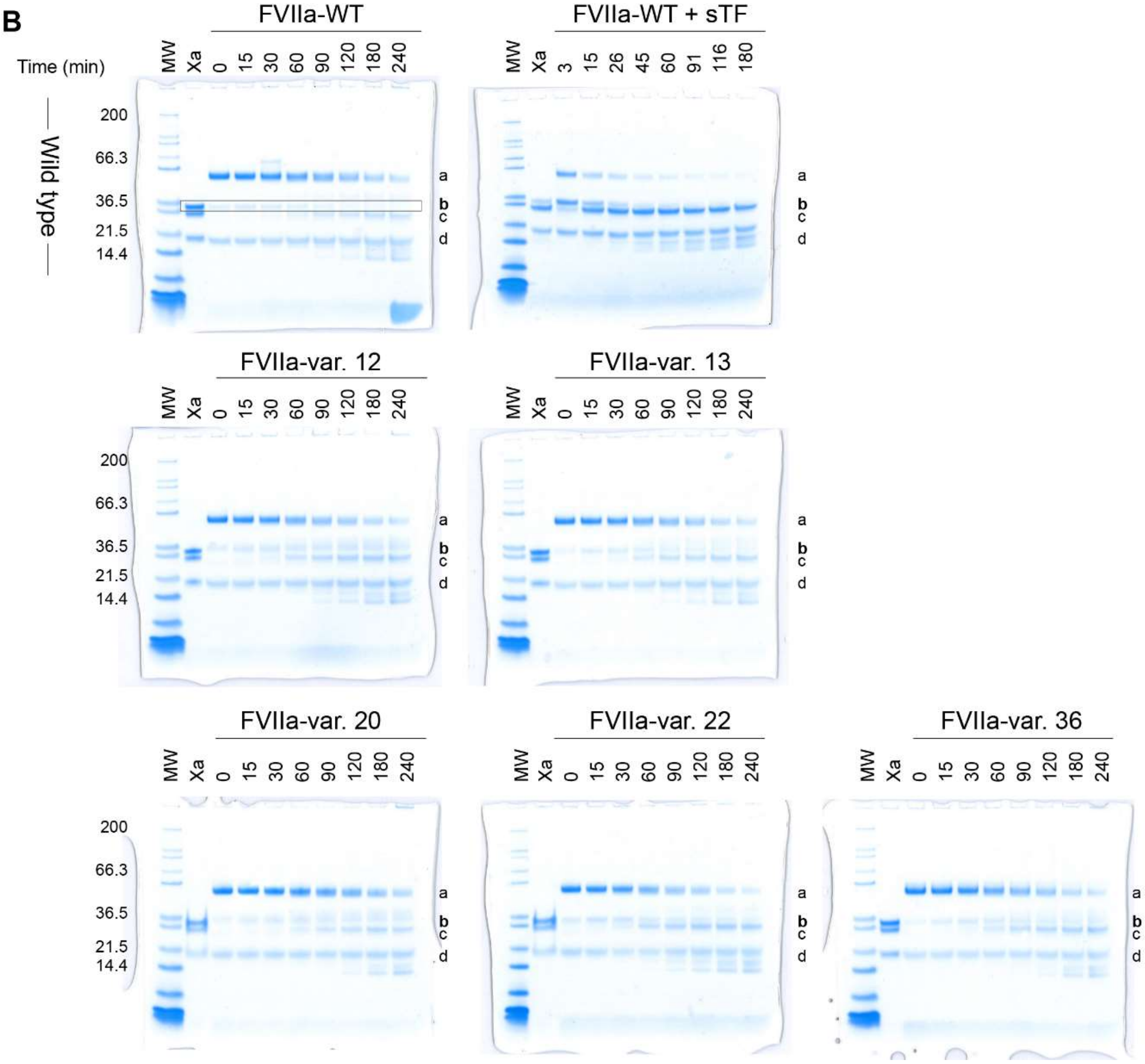

Supplemental Figure S8 FX-activation kinetics of selected FVIIa-variants visualized by SDS-PAGE. **(A)** Initial FX-activation rate estimated by gel densitometric analysis of FXa HCβ (c-band) product band on gels. **(B)** SDS-PAGE analysis of FX-activation by selected FVIIa-variants. MW = molecular weight marker, Xa = FXa control. Major protein bands observed on gel: a = FX HC, b = FXa HCα, c = FXa HCβ and d = FX/FXa LC
